# Reporting quality of randomized controlled trials evaluating non-vitamin K oral anticoagulants in atrial fibrillation: a systematic review

**DOI:** 10.1186/s12872-023-03258-z

**Published:** 2023-05-03

**Authors:** YueGuang Yang, YuBo Han, GuoLiang Zou, YanBo Sui, Juan Jin, Li Liu

**Affiliations:** 1 Heilongjiang University of Chinese Medicine, Harbin, P. R. China; 2 The First Department of Cardiovascular, First Affiliated Hospital, Heilongjiang University of Chinese Medicine, 26 Heping Road, Xiangfang, Harbin, Heilongjiang 150040 P.R. China

**Keywords:** Randomized controlled trial, Atrial fibrillation, Non-vitamin K oral anticoagulants consolidated, Reporting quality, Standards of reporting trials statement

## Abstract

**Background:**

Randomized controlled trials (RCTs) are subject to bias if they lack methodological quality. Furthermore, optimal and transparent reporting of RCT findings aids their critical appraisal and interpretation. This study aimed to comprehensively evaluate the report quality of RCTs of non-vitamin K oral anticoagulants (NOACs) for the treatment of atrial fibrillation (AF) and to analyze the factors influencing the quality.

**Methods:**

By searching PubMed, Embase, Web of Science, and Cochrane Library databases RCTs published from inception to 2022 evaluating the efficacy of NOACs on AF were collected. By using the 2010 Consolidated Standards for Reporting Tests (CONSORT) statement, the overall quality of each report was assessed.

**Results:**

Sixty-two RCTs were retrieved in this study. The median of overall quality score in 2010 was 14 (range: 8.5–20). The extent of compliance with the Consolidated Standards of Reporting Trials reporting guideline differed substantially across items: 9 items were reported adequately (more than 90%), and 3 were reported adequately in less than 10% of trials. Multivariate linear regression analysis showed that the higher reporting scores were associated with higher journal impact factor (*P* = 0.01), international collaboration (*P* < 0.01), and Sources of trial funding (*P *= 0.02).

**Conclusions:**

Although a large number of randomized controlled trials of NOACs for the treatment of AF were published after the CONSORT statement in 2010, the overall quality is still not satisfactory, thus weakening their potential utility and may mislead clinical decisions. This survey provides the first hint for researchers conducting trials of NOACs for AF to improve the quality of reports and to actively apply the CONSORT statement.

**Supplementary Information:**

The online version contains supplementary material available at 10.1186/s12872-023-03258-z.

## Introduction

Randomized Controlled Trial (RCT) is an optimal design to evaluate the effect of treatment [1]. High-quality RCTs can effectively reduce bias and provide evidence for clinical practice, while poor RCTs may adversely affect routine clinical practice [2]. Since high-quality reporting helps readers critically evaluate the quality of trials, it is of great significance to identify major quality predictions for such reporting. The Consolidated Test Reporting Standard (CONSORT) is a set of recommendations based on the least evidence, including checklists and flowcharts for RCT reports, which can help to judge the standardization and repeatability of RCTs [3]. Its purpose is to promote transparent and comprehensive trials reporting and to assist in the critical evaluation and interpretation of trials.

Atrial fibrillation (AF), one of the most common arrhythmias [4], is associated with a high rate of disability and mortality [5], increasing the risks of stroke, embolism, and death [6–8]. With the aging of the population, the incidence of AF is also on the increase year by year [9]. Therefore, the early treatment of AF is quite important.

Drug is administered primarily for the prevention of stroke and systemic embolism in the treatment of AF In the guidelines, warfarin has long been recommended as the preferred anticoagulation agent for AF, but it is subject to certain limitations and requires long-term and frequent laboratory tests [10]. As a new type of anticoagulant, the non-vitamin K oral anticoagulant (NOAC) works by preventing thrombin production and inhibiting factor Xa, which eliminates the need for long-term monitoring. Research has shown that NOACs are more effective and safer than warfarin [11–14], making it a promising replacement for warfarin in the future [15]. Therefore, it is particularly important to determine the accuracy of the RCTs of NOACs for AF.

This study aimed to evaluate the overall reporting quality and identify some essential issues of published RCTs of NOACs for AF based on the CONSORT statement and to analyze possible related causes, so as to provide reliable evidence for subsequent related studies and meta-analyses.

## Methods

This systematic review was reported according to the PRISMA statement [16].

### Search strategy

A comprehensive literature search was conducted to identify humans’ prospective randomized controlled trials. Through a manual trial search on advanced PubMed, Embase, Web of Science, and Cochrane Library databases all journal articles published in English as of September 2022 were obtained. The survey was accomplished in duplicate. Also, the final search standards were " atrial fibrillation " and “one of the four NOACs–apixaban, dabigatran, edoxaban, and rivaroxaban”. There was no limit to the year of publication, to the sample size or to the type of research (advantages, equivalence, non-disadvantages).

### Inclusion and exclusion criteria

Studies meeting the following criteria were included in the study: (1) randomized controlled trials; (2) study subjects involved patients with AF; (3) interventions related to one of the four NOACs–apixaban, dabigatran, edoxaban, and rivaroxaban. The exclusion criteria were as follows: (1) duplicate publications; (2) animal experiments, and subsequently withdrawn publications; (3) abstract or full text not available.

### Assessment of the reporting quality

Two reviewers (YYG and HYB) independently followed a standardized evaluation checklist to review and extract data from eligible articles. The 2010 CONSORT criteria were adopted. Each item on the criteria checklist was scored, from satisfied to dissatisfied. Any divergence between these two reviewers about these articles would be resolved by a third reviewer(ZGL). Cohen’s kappa will be calculated to assess consistency between reviewer ratings(0.45 to 0.81). A total of 25 spouse statements were revised in 2010. Among them, 12 items were separated into two parts (37 items in total). 1 point was assigned if item was reported, 0.5 points was assigned if sub-item was reported and 0 point was assigned if these items were not reported. An overall CONSORT score (score range 0 to25) was calculated by totaling the scores of all 37 quality items.

### Statistical analysis

By using SPSS Version 24.0, descriptive statistics were generated, covering CONSORT (0–25) based on median and interquartile ranges. Categorical data are presented as a number (n) and percent (%), and the reporting score is recorded as mean and standard deviations (SD). Independence t-test and one-way analysis of variance (ANOVA) are used to compare the differences of general features, as the data are consistent with the homogeneity and normality of variance. Multi-distant linear regression analysis was used to explore the relationship between influencing factors and report quality. Potential predictors were coded as follows: Year of publication: 2001–2010 = 1, 2011–2022 = 2; Region in which trials were: Asia = 1, Europe and North America = 2; Sources of trial funding: Funding not reported = 1, Completely funded by industry = 2, Government and industry = 3, Government/foundation = 4; Journal impact factor:< 4 = 1, 4–10 = 2, ≥ 10 = 3; Sample size;≤200 = 1, 200–400 = 2, ≥ 400 = 3; International collaboration: no = 1, yes = 2. For all analyses, the statistical significance level was set at *P* < 0.05.

## Results

### Search results

**Fig. 1:**
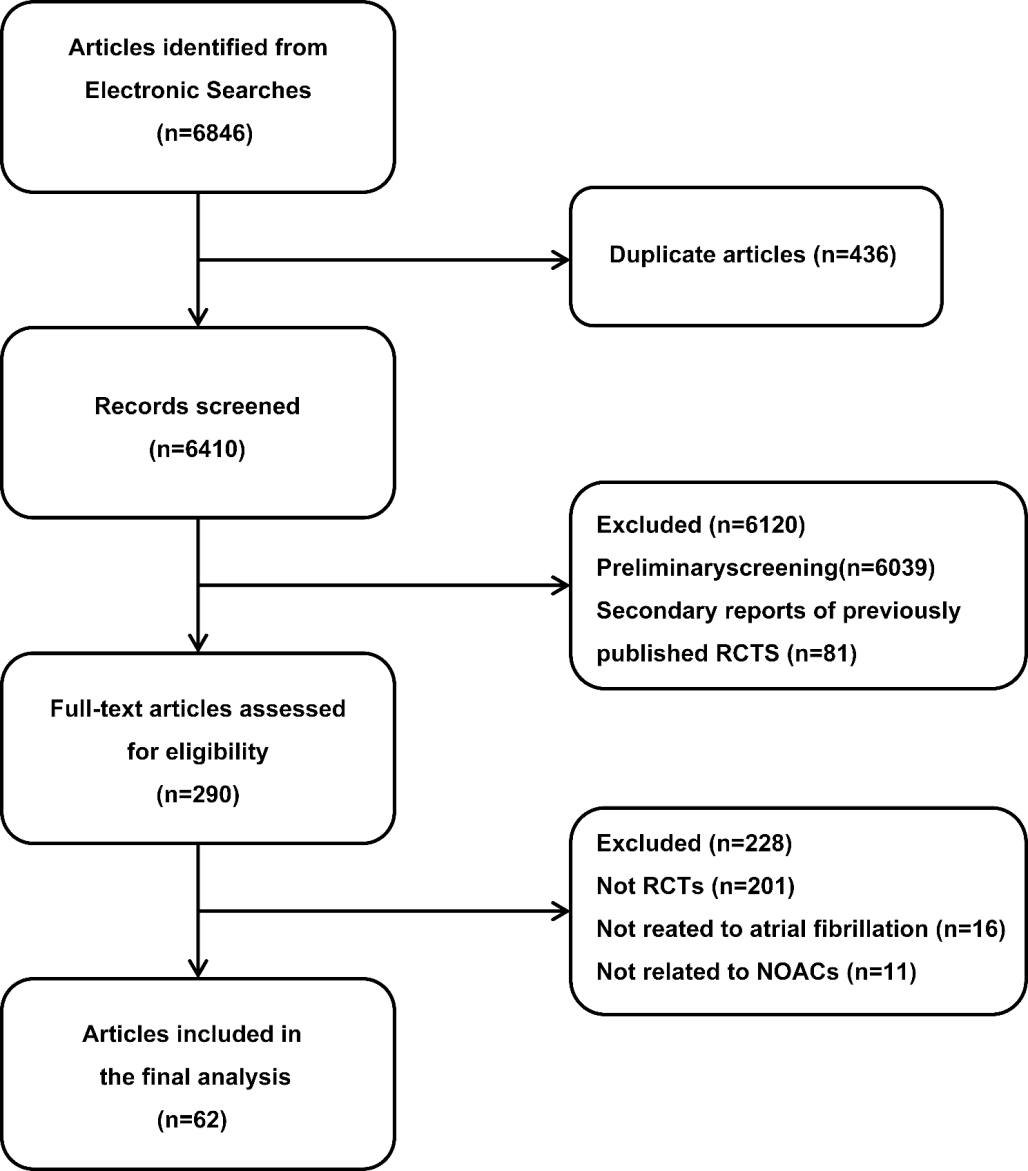
Selection of randomized clinical trials in the systematic review

According to the literature search results, 290 studies (Fig. 1) were found. Among them, 16 studies were removed as they were unrelated to AF, and 201 studies were excluded because they were not RCTs. Also, 11 studies were ruled out since they didn’t include NOACs. In the end, 62 studies were included, which were also parallel controlled studies.

### Characteristics of the included studies

Table 1 showed the Characteristics of RCTs included in this study. Most articles were published after 2010, accounting for 93.54%. 58.06% of the trials were conducted in Europe and North America. 46.77% of the trials were completely funded by the industry. 45.16% of the trials were published in journals with an impact factor greater than 10. 37 (59.86%) trials were compared with warfarin. The sample size of 50% trials is more than 400. Most of the trials (56.45%) did not involve international cooperation. In addition, the differences in consort scores of “Sources of trial funding” (*P* = 0.040), “Journal impact factor”(*P* < 0.01), “Sample size”(*P* < 0.01) and “International collaboration”(*P* = 0.017) were statistically significant.

**Table 1:** Trial characteristics

Characteristic	No. Of studies	CONSORT sore(mean ± SD)	F/t	*P*-vale
Year of publication				
2001–2010	4(6.45%)	15.13 ± 3.45	0.934	0.354
2011–2022	58(93.54%)	13.85 ± 2.58		
Region in which trials were conducted				
Asia	26(41.93%)	13.85 ± 2.34	-1.413	0.163
Europe and North America	36(58.06%)	14.33 ± 2.79		
Sources of trial funding				
Government/foundation	10(16.13%)	15.00 ± 3.09	2.948	0.040
Government and industry	5(8.06%)	14.70 ± 2.28		
Completely funded by industry	29(46.77%)	13.95 ± 2.73		
Funding not reported	18(29.03%)	12.28 ± 2.14		
Journal impact factor				
< 4	20(32.26%)	12.58 ± 2.17	20.418	< 0.01
4–10	14(22.58%)	12.21 ± 2.32		
> 10	28(45.16%)	15.77 ± 2.63		
The control group interventions				
Warfarin	37(59.68%)	14.07 ± 2.62	0.478	0.634
Others	25(40.32%)	13.74 ± 2.69		
Sample size				
< 200	22(35.48%)	12.57 ± 2.22	7.127	< 0.01
200–400	9(14.52%)	13.61 ± 2.06		
> 400	31(50%)	13.94 ± 2.63		
International collaboration				
Yes	27(43.55%)	14.83 ± 2.71	2.457	0.017
No	35(56.45%)	13.24 ± 2.38		

### Reporting of all items

**Fig. 2:**
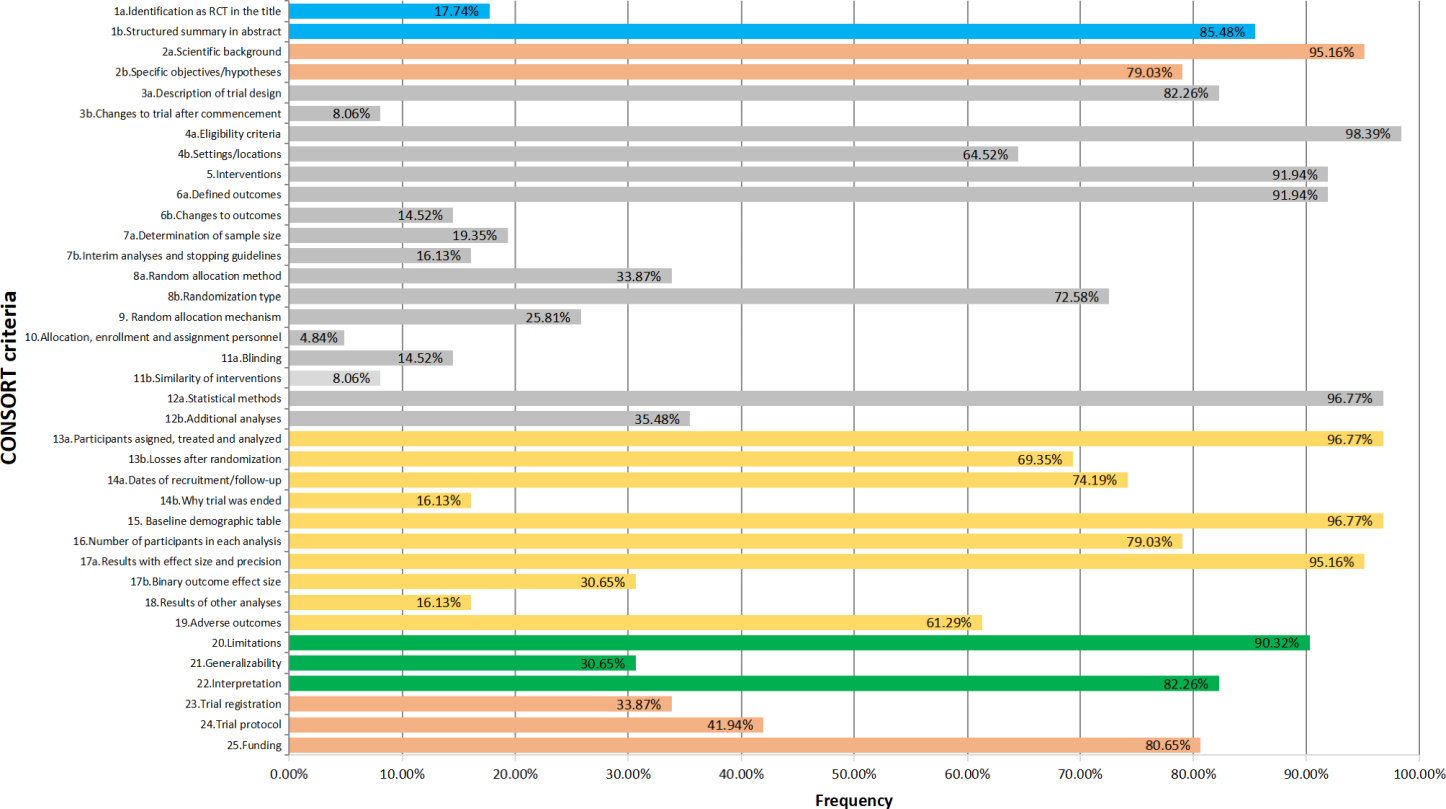
Overall Quality of Reporting: Rating Based on Items in the 2010 CONSORT Statement (n = 62)

**Fig. 3:**
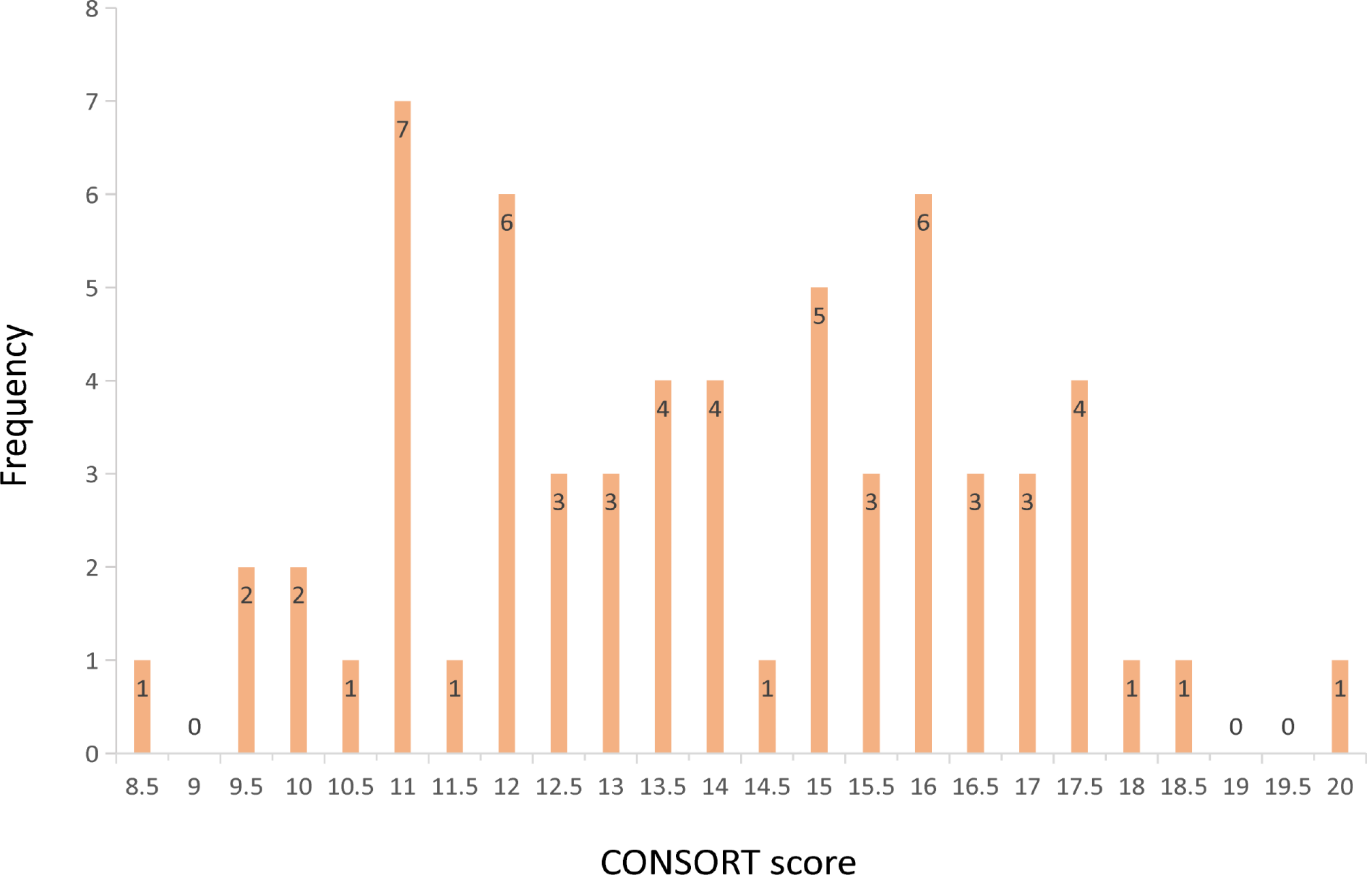
The frequency bar graphs of all researched OQS (n = 62)

Figure 2 showed the research characteristics and descriptive results. Overall, the average reporting rate of all items was 55.45%. Specifically, Table 2 displays that 6 items were fully reported (> 95%) and 5 items were poorly reported (< 15%). The median of overall quality score in 2010 was 14 (range: 8.5–20). Figure 3 shows the frequency bar graphs of all researched OQS.

**Table 2:** Report details for selected items

Item Description	Frequency (n)	Adherence (%)
Scientific background	59/62	95.16
Changes to trial after commencement	5/62	8.06
Eligibility criteria	61/62	98.39
Changes to outcomes	9/62	14.52
Allocation, enrollment and assignment personnel	3/62	4.84
Blinding	9/62	14.52
Similarity of interventions	5/62	8.06
Statistical methods	60/62	96.77
Participants assigned, treated and analyzed	60/62	96.77
Baseline demographic table	60/62	96.77
Results with effect size and precision	59/62	95.16

### Factors associated with reporting quality

Table 3 shows the results of the linear regression modeling. Six independent variables are input into the multiple linear regression model, among which the Sources of trial funding(*P* = 0.02), journal impact factor (*P* = 0.01), and international collaboration (*P* < 0.01) as noticeable predictors of the overall CONSORT score.

**Table 3:** Factors associated with key elements of the CONSORT guidelines

Characteristics	Mean OQS (95% CI)	Standardized Coefficients		Standardized Coefficients Beta	t	Estimate 95% CI		*p*-vale
		B	SE			Lower	Upper	
Constant		8.35	2.41		3.47	3.53	13.18	0.00
Year of publication		0.04	1.03	0.00	0.04	-2.03	2.10	0.97
2001–2010	15.12 (10 to 17.5)							
2011–2022	13.94(8.5 to 20)							
Region in which trials were		-0.74	0.55	-0.14	-1.33	-1.85	0.37	0.19
Asia	13.94 (9.5 to 18.5)							
Europe and North America	13.92 (8.5 to 20)							
Sources of trial funding		0.59	0.25	0.24	2.34	0.08	1.09	0.02
Not reported	13.51 (10 to 18)							
Industry	13.71 (8.5 to 20)							
Government and industry	13.82 (11 to 16.5)							
Government	14.09 (9.5 to 18.5)							
Journal impact factor		1.07	0.36	0.36	2.95	0.34	1.79	0.01
< 4	13.13 (8.5 to 17)							
4–10	13.86 (9.5 to 17.5)							
≥ 10	14.08 (12 to 20)							
Sample size		-0.04	0.34	-0.01	-0.11	-0.72	0.65	0.91
≤ 200	13.60 (8.5 to 17)							
200–400	13.59 (11 to 17.5)							
≥ 400	14.13 (9.5 to 20)							
International collaboration		2.18	0.62	0.42	3.54	0.95	3.42	< 0.01
No	13.17 (8.5 to 20)							
Yes	13.94 (9.5 to 18.5)							

## Discussion

In order to ensure accuracy and consistency in the reporting of RCTs, it is crucial that they are presented in an open and transparent manner, adhering strictly to the CONSORT guidelines. Failure to do so may result in overestimation of therapeutic efficacy and conflicting conclusions. [17–20]. Since 1996, the reporting quality of RCTs has improved dramatically when the CONSORT was launched [21], particularly those trials concerning drugs [22, 23]. CONSORT was updated in 2001 and 2010 [24, 25], respectively.

Unfortunately, although most RCTs of NOACs in AF have been published since the revision of 2010 CONSORT statement, the overall reporting quality was still unsatisfactory, with an overall average reporting rate of 55.45%, which is similar to the findings in the fields of lung cancer, and COVID-19. In addition, fewer project reports were added to or redefined in the 2010 revision compared with the 2001 CONSORT statement.

In this study, it was found that some items and measures of clinical characteristics had a high reporting rate of results, such as items 2a, 4a, 12a, 13a, 15, 17a, (95.16%, 98.39%, 96.77%, 96.77%, 96.77%,95.16%), which put emphasis on the theoretical background of the trials, eligibility criteria, statistical methods, participants assigned, baseline demographic table, and results with effect size and precision. However, the reporting rates of Changes to trial after commencement, Changes to outcomes, Interim analyses and stopping guidelines, Allocation, enrollment and assignment personnel, Blinding, Similarity of interventions, and Dates of recruitment (items 3b,6b,7b,10,11a,11b,14b) were only 8.06%, 14.52%, 16.13%, 4.94%, 14.52%, 8.06% and 16.13%, respectively. The results are worrying because the missing reporting items are also an important part of the details of the trial, and they are equally noteworthy. This finding was consistent with earlier research [26–28].

The results are worrying because the missing reporting items are also an important part of the details of the trial, and they are equally noteworthy. Inappropriate methods of randomization can lead to selection and confounding bias [29], and most trials that lack details of the randomization procedure provided also have problems with randomization-related bias, as well as influencing the reader’s assessment of the risk of selection bias [30]. In RCTs, blinding is used to avoid subjective bias. However, inappropriate blinding can increase the risk of bias and exaggerated results [30–32]. Some of the RCTs of NOACs for AF included in this study were not completely randomized trials per se, but used district group randomization, and patients in different groups may have different clinical characteristics and risk factors, which may introduce some bias and thus affect the reliability of the results [33–37]. Similarly, the lack of reporting of the way in which warfarin blinding was performed (blinded, unblended, masked) in some of the trials that were compared with warfarin may have introduced bias in the trial results [35, 38–41]. These should be discussed in the report. Reporting of significant events (patients lost to follow-up, treatment adherence, unbinding, and cross-contamination) after trial initiation is important, and these factors may also have an impact on trial results [42]. Especially in trials related to NOACs, the reporting of bleeding events plays an important role in the reliability of trial results. Secondly, the reporting of abnormal events is also essential in clinical trials, as these events may affect the validity and reliability of trial results [43]. For example, in the RE-LY trial, the reporting of myocardial infarction rate is an important issue because it may affect the interpretation and promotion of the trial results [44–46]. Therefore, accurate and clear event reporting in clinical trials is important and helps to increase the accuracy and reliability of trial results. The reason for these problems may be that the clinical features of RCTs are considered more important and engaging since many authors are also clinicians.

The reason for these problems may be that the clinical features of RCTs are considered more important and attractive because many authors are also clinicians. Here, it is suggested that the design of experimental methods should be carried out through full cooperation of scientists and clinicians. In addition, it is possibly lied in that most trials had developed detailed plans and remedial measures before they started, and that the trials proceeded smoothly without errors or RCTs with early termination or negative results are often rejected for publication [47]. For the sake of simplicity, the detailed test procedures of some articles cannot be fully reported, thus affecting the quality of the report. Experiments have shown a close relationship between inadequately informed trials and poorly designed or conducted tests [48, 49]. Hence, it is suggested that journals can revise their requirements for word count, especially for RCT, or offer online resources that facilitate authors to make these key omissions.

This study shows that journal impact factors, experimental Sources of trial funding and international collaboration are also important factors in the quality of RCTs reporting. Better reporting quality is associated with higher journal impact factors, international cooperation and high sample size, which is similar to a previous study [50, 51]. This may be because journals with higher impact factors have higher requirements for papers [52], and many countries participate in cooperative experiments with more perfect and detailed experimental designs. We believe that the potential reason for the poor quality of RCT reports is that some journals do not evaluate articles strictly according to the CONSORT statement, so we suggest that journal editors and peer reviewers should strictly judge the completeness and accuracy of the tests according to the CONSORT statement when reviewing the RCT. Meanwhile, the journal should also increase the promotion of CONSORT statement, calling on researchers to refer to CONSORT statement to design RCT experiments and write articles.

Nevertheless, this study is also plagued by several limitations. The release time of NOACs is relatively short; there are few RCTs on it; the number of included trials is limited. Besides, each CONSORT item is assigned with an equal weight, which may overemphasize some less important things. In addition, some of the item requirements in the CONSORT statement are very complex; although it provides explanatory notes, and we have designed and tried data collection templates prior to data collection, including detailed reporting requirements for specific items (such as allocation, hiding, blocking or setting and location), it is still impossible to avoid subjective factors during grading.

## Conclusion

According to the data collected herein, there is currently a lack of clinical randomized controlled studies of NOACs in the treatment of atrial fibrillation. The quality of the existing articles needs to be improved. However, for confirming the effect of treatment, RCT is still recommended by this study as the preferred design in an ideal environment. In the future, more comprehensive efforts shall be made to increase the awareness of readers, reviewers, and editors on these issues. At the same time, the quality and accuracy improvement of such research conclusions shall also be focused on to guarantee the credibility of the evidence that they have offered.

## Electronic supplementary material

Below is the link to the electronic supplementary material.

Supplementary Material 1: Supplementary Appendix to the 62 RCTs included in this systematic review

## Data Availability

The datasets used and/or analyzed during the current study are available from the corresponding author on reasonable request.
