# Supplementary material for: Reporting quality of randomized controlled trials evaluating non-vitamin K oral anticoagulants in atrial fibrillation: a systematic review
Source: BMC Cardiovasc Disord. 2023 May 3;23:229. doi: 10.1186/s12872-023-03258-z (PMC10155658; doi:10.1186/s12872-023-03258-z)
Supplement: Supplementary file 1 — Supplementary Material 1: Supplementary Appendix to the 62 RCTs included in this systematic review [file 12872_2023_3258_MOESM1_ESM.pdf]

Supplementary Appendix to the 62 RCTs included in the systematic review: Reporting Quality of Randomized Controlled Trials Evaluating Non-Vitamin K Oral Anticoagulants in Atrial Fibrillation

1. Connolly SJ, Ezekowitz MD, Yusuf S, Eikelboom J, Oldgren J, Parekh A, Pogue J, Reilly PA, Themeles E, Varrone J *et al*: **Dabigatran versus warfarin in patients with atrial fibrillation**. *The New England journal of medicine* 2009, **361**(12):1139-1151.
2. Guimarães HP, Lopes RD, de Barros ESPGM, Liporace IL, Sampaio RO, Tarasoutchi F, Hoffmann-Filho CR, de Lemos Soares Patriota R, Leiria TLL, Lamprea D *et al*: **Rivaroxaban in Patients with Atrial Fibrillation and a Bioprosthetic Mitral Valve**. *The New England journal of medicine* 2020, **383**(22):2117-2126.
3. Yasuda S, Kaikita K, Akao M, Ako J, Matoba T, Nakamura M, Miyauchi K, Hagiwara N, Kimura K, Hirayama A *et al*: **Antithrombotic Therapy for Atrial Fibrillation with Stable Coronary Disease**. *The New England journal of medicine* 2019, **381**(12):1103-1113.
4. Lopes RD, Heizer G, Aronson R, Vora AN, Massaro T, Mehran R, Goodman SG, Windecker S, Darius H, Li J *et al*: **Antithrombotic Therapy after Acute Coronary Syndrome or PCI in Atrial Fibrillation**. *The New England journal of medicine* 2019, **380**(16):1509-1524.
5. Giugliano RP, Ruff CT, Braunwald E, Murphy SA, Wiviott SD, Halperin JL, Waldo AL, Ezekowitz MD, Weitz JI, špinar J *et al*: **Edoxaban versus warfarin in patients with atrial fibrillation**. *The New England journal of medicine* 2013, **369**(22):2093-2104.
6. Gibson CM, Mehran R, Bode C, Halperin J, Verheugt FW, Wildgoose P, Birmingham M, Ianus J, Burton P, van Eickels M *et al*: **Prevention of Bleeding in Patients with Atrial Fibrillation Undergoing PCI**. *The New England journal of medicine* 2016, **375**(25):2423-2434.
7. Cannon CP, Bhatt DL, Oldgren J, Lip GYH, Ellis SG, Kimura T, Maeng M, Merkely B, Zeymer U, Gropper S *et al*: **Dual Antithrombotic Therapy with Dabigatran after PCI in Atrial Fibrillation**. *The New England journal of medicine* 2017, **377**(16):1513-1524.
8. Cannon CP, Bhatt DL, Oldgren J, Lip GYH, Ellis SG, Kimura T, Maeng M, Merkely B, Zeymer U, Gropper S *et al*: **Dual Antithrombotic Therapy with Dabigatran after PCI in Atrial Fibrillation**. *New England Journal of Medicine* 2017, **377**(16):1513-1524.
9. Gibson CM, Mehran R, Bode C, Halperin J, Verheugt F, Wildgoose P, van Eickels M, Lip GY, Cohen M, Husted S *et al*: **An open-label, randomized, controlled, multicenter study exploring two treatment strategies of rivaroxaban and a dose-adjusted oral vitamin K antagonist treatment strategy in subjects with atrial fibrillation who undergo percutaneous coronary intervention (PIONEER AF-PCI)**. *Am Heart J* 2015, **169**(4):472-478.e475.
10. Connolly SJ, Eikelboom J, Joyner C, Diener HC, Hart R, Golitsyn S, Flaker G, Avezum A, Hohnloser SH, Diaz R *et al*: **Apixaban in patients with atrial fibrillation**. *The New England journal of medicine* 2011, **364**(9):806-817.
11. Calkins H, Willems S, Gerstenfeld EP, Verma A, Schilling R, Hohnloser SH, Okumura K,

Serota H, Nordaby M, Guiver K *et al*: **Uninterrupted Dabigatran versus Warfarin for Ablation in Atrial Fibrillation**. *The New England journal of medicine* 2017, **376**(17):1627-1636.

12. De Vriese AS, Caluwé R, Van Der Meersch H, De Boeck K, De Bacquer D: **Safety and Efficacy of Vitamin K Antagonists versus Rivaroxaban in Hemodialysis Patients with Atrial Fibrillation: A Multicenter Randomized Controlled Trial**. *Journal of the American Society of Nephrology : JASN* 2021, **32**(6):1474-1483.
13. Ezekowitz MD, Pollack CV, Jr., Halperin JL, England RD, VanPelt Nguyen S, Spahr J, Sudworth M, Cater NB, Breazna A, Oldgren J *et al*: **Apixaban compared to heparin/vitamin K antagonist in patients with atrial fibrillation scheduled for cardioversion: the EMANATE trial**. *European heart journal* 2018, **39**(32):2959-2971.
14. Patel MR, Mahaffey KW, Garg J, Pan G, Singer DE, Hacke W, Breithardt G, Halperin JL, Hankey GJ, Piccini JP *et al*: **Rivaroxaban versus warfarin in nonvalvular atrial fibrillation**. *The New England journal of medicine* 2011, **365**(10):883-891.
15. Osmancik P, Herman D, Neuzil P, Hala P, Taborsky M, Kala P, Poloczek M, Stasek J, Haman L, Branny M *et al*: **Left Atrial Appendage Closure Versus Direct Oral Anticoagulants in High-Risk Patients With Atrial Fibrillation**. *Journal of the American College of Cardiology* 2020, **75**(25):3122-3135.
16. Cappato R, Marchlinski FE, Hohnloser SH, Naccarelli GV, Xiang J, Wilber DJ, Ma CS, Hess S, Wells DS, Juang G *et al*: **Uninterrupted rivaroxaban vs. uninterrupted vitamin K antagonists for catheter ablation in non-valvular atrial fibrillation**. *European heart journal* 2015, **36**(28):1805-1811.
17. Hohnloser SH, Camm J, Cappato R, Diener HC, Heidbüchel H, Mont L, Morillo CA, Abozguia K, Grimaldi M, Rauer H *et al*: **Uninterrupted edoxaban vs. vitamin K antagonists for ablation of atrial fibrillation: the ELIMINATE-AF trial**. *European heart journal* 2019, **40**(36):3013-3021.
18. Granger CB, Alexander JH, McMurray JJ, Lopes RD, Hylek EM, Hanna M, Al-Khalidi HR, Ansell J, Atar D, Avezum A *et al*: **Apixaban versus warfarin in patients with atrial fibrillation**. *The New England journal of medicine* 2011, **365**(11):981-992.
19. Cappato R, Ezekowitz MD, Klein AL, Camm AJ, Ma CS, Le Heuzey JY, Talajic M, Scanavacca M, Vardas PE, Kirchhof P *et al*: **Rivaroxaban vs. vitamin K antagonists for cardioversion in atrial fibrillation**. *European heart journal* 2014, **35**(47):3346-3355.
20. Hori M, Matsumoto M, Tanahashi N, Momomura S, Uchiyama S, Goto S, Izumi T, Koretsune Y, Kajikawa M, Kato M *et al*: **Rivaroxaban vs. warfarin in Japanese patients with atrial fibrillation – the J-ROCKET AF study –**. *Circulation journal : official journal of the Japanese Circulation Society* 2012, **76**(9):2104-2111.
21. Durães AR, de Souza Roriz P, de Almeida Nunes B, Albuquerque FP, de Bulhões FV, de Souza Fernandes AM, Aras R: **Dabigatran Versus Warfarin After Bioprosthesis Valve Replacement for the Management of Atrial Fibrillation Postoperatively: DAWA Pilot Study**. *Drugs in R&D* 2016, **16**(2):149-154.
22. Kirchhof P, Haeusler KG, Blank B, De Bono J, Callans D, Elvan A, Fetsch T, Van Gelder IC, Gentlesk P, Grimaldi M *et al*: **Apixaban in patients at risk of stroke undergoing atrial fibrillation ablation**. *European heart journal* 2018, **39**(32):2942-2955.
23. Maeng M, Steg PG, Bhatt DL, Hohnloser SH, Nordaby M, Miede C, Kimura T, Lip GYH,

Oldgren J, Ten Berg JM *et al*: **Dabigatran Dual Therapy Versus Warfarin Triple Therapy Post-PCI in Patients With Atrial Fibrillation and Diabetes**. *JACC Cardiovascular interventions* 2019, **12**(23):2346-2355.

24. Hong KS, Kwon SU, Lee SH, Lee JS, Kim YJ, Song TJ, Kim YD, Park MS, Kim EG, Cha JK *et al*: **Rivaroxaban vs Warfarin Sodium in the Ultra-Early Period After Atrial Fibrillation-Related Mild Ischemic Stroke: A Randomized Clinical Trial**. *JAMA neurology* 2017, **74**(10):1206-1215.
25. Nogami A, Harada T, Sekiguchi Y, Otani R, Yoshida Y, Yoshida K, Nakano Y, Nuruki N, Nakahara S, Goya M *et al*: **Safety and Efficacy of Minimally Interrupted Dabigatran vs Uninterrupted Warfarin Therapy in Adults Undergoing Atrial Fibrillation Catheter Ablation: A Randomized Clinical Trial**. *JAMA network open* 2019, **2**(4):e191994.
26. Birnie DH, Healey JS, Wells GA, Ayala-Paredes F, Coutu B, Sumner GL, Becker G, Verma A, Philippon F, Kalfon E *et al*: **Continued vs. interrupted direct oral anticoagulants at the time of device surgery, in patients with moderate to high risk of arterial thrombo-embolic events (BRUISE CONTROL-2)**. *European heart journal* 2018, **39**(44):3973-3979.
27. Reynolds MR, Allison JS, Natale A, Weisberg IL, Ellenbogen KA, Richards M, Hsieh WH, Sutherland J, Cannon CP: **A Prospective Randomized Trial of Apixaban Dosing During Atrial Fibrillation Ablation: The AEIOU Trial**. *JACC Clinical electrophysiology* 2018, **4**(5):580-588.
28. Ke HH, He Y, Lv XW, Zhang EH, Wei Z, Li JY: **Efficacy and safety of rivaroxaban on the resolution of left atrial/left atrial appendage thrombus in nonvalvular atrial fibrillation patients**. *Journal of thrombosis and thrombolysis* 2019, **48**(2):270-276.
29. Weitz JI, Connolly SJ, Patel I, Salazar D, Rohatagi S, Mendell J, Kastrissios H, Jin J, Kunitada S: **Randomised, parallel-group, multicentre, multinational phase 2 study comparing edoxaban, an oral factor Xa inhibitor, with warfarin for stroke prevention in patients with atrial fibrillation**. *Thrombosis and haemostasis* 2010, **104**(3):633-641.
30. Yu HT, Shim J, Park J, Kim TH, Uhm JS, Kim JY, Joung B, Lee MH, Kim YH, Pak HN: **When is it appropriate to stop non-vitamin K antagonist oral anticoagulants before catheter ablation of atrial fibrillation? A multicentre prospective randomized study**. *European heart journal* 2019, **40**(19):1531-1537.
31. Bertaglia E, Anselmino M, Zorzi A, Russo V, Toso E, Peruzza F, Rapacciuolo A, Migliore F, Gaita F, Cucchini U *et al*: **NOACs and atrial fibrillation: Incidence and predictors of left atrial thrombus in the real world**. *International journal of cardiology* 2017, **249**:179-183.
32. Yamaji H, Murakami T, Hina K, Higashiya S, Kawamura H, Murakami M, Kamikawa S, Hirohata S, Kusachi S: **Activated clotting time on the day of atrial fibrillation ablation for minimally interrupted and uninterrupted direct oral anticoagulation therapy: Sequential changes, differences among direct oral anticoagulants, and ablation safety outcomes**. *Journal of cardiovascular electrophysiology* 2019, **30**(12):2823-2833.
33. Shah RR, Pillai A, Schafer P, Meggo D, McElderry T, Plumb V, Yamada T, Kumar V, Doppalapudi H, Gunter A *et al*: **Safety and Efficacy of Uninterrupted Apixaban Therapy Versus Warfarin During Atrial Fibrillation Ablation**. *The American journal of cardiology* 2017, **120**(3):404-407.

34. Zhu J, Gao RJ, Liu Q, Jiang RH, Yu L, Sun YX, Zhang P, Lin JW, Ye Y, Zhang ZW *et al*: **Metabolic benefits of rivaroxaban in non-valvular atrial fibrillation patients after radiofrequency catheter ablation**. *Journal of Zhejiang University Science B* 2017, **18**(11):946-954.
35. Ando M, Inden Y, Yoshida Y, Sairaku A, Yanagisawa S, Suzuki H, Watanabe R, Takenaka M, Maeda M, Murohara T: **Differences in prothrombotic response between the uninterrupted and interrupted apixaban therapies in patients undergoing cryoballoon ablation for paroxysmal atrial fibrillation: a randomized controlled study**. *Heart and vessels* 2019, **34**(9):1533-1541.
36. Yamaji H, Murakami T, Hina K, Higashiya S, Kawamura H, Murakami M, Kamikawa S, Komtasubara I, Kusachi S: **Adequate Initial Heparin Dosage for Atrial Fibrillation Ablation in Patients Receiving Non-Vitamin K Antagonist Oral Anticoagulants**. *Clinical drug investigation* 2016, **36**(10):837-848.
37. Nagao T, Suzuki H, Matsunaga S, Nishikawa Y, Harada K, Mamiya K, Shinoda N, Harada K, Kato M, Marui N *et al*: **Impact of periprocedural anticoagulation therapy on the incidence of silent stroke after atrial fibrillation ablation in patients receiving direct oral anticoagulants: uninterrupted vs. interrupted by one dose strategy**. *Europace : European pacing, arrhythmias, and cardiac electrophysiology : journal of the working groups on cardiac pacing, arrhythmias, and cardiac cellular electrophysiology of the European Society of Cardiology* 2019, **21**(4):590-597.
38. Connolly SJ, Eikelboom J, Dorian P, Hohnloser SH, Gretler DD, Sinha U, Ezekowitz MD: **Betrixaban compared with warfarin in patients with atrial fibrillation: results of a phase 2, randomized, dose-ranging study (Explore-Xa)**. *European heart journal* 2013, **34**(20):1498-1505.
39. Eikelboom JW, Connolly SJ, Gao P, Paolasso E, De Caterina R, Husted S, O'Donnell M, Yusuf S, Hart RG: **Stroke risk and efficacy of apixaban in atrial fibrillation patients with moderate chronic kidney disease**. *Journal of stroke and cerebrovascular diseases : the official journal of National Stroke Association* 2012, **21**(6):429-435.
40. Ogawa S, Shinohara Y, Kanmuri K: **Safety and efficacy of the oral direct factor xa inhibitor apixaban in Japanese patients with non-valvular atrial fibrillation. -The ARISTOTLE-J study**. *Circulation journal : official journal of the Japanese Circulation Society* 2011, **75**(8):1852-1859.
41. Kimura T, Kashimura S, Nishiyama T, Katsumata Y, Inagawa K, Ikegami Y, Nishiyama N, Fukumoto K, Tanimoto Y, Aizawa Y *et al*: **Asymptomatic Cerebral Infarction During Catheter Ablation for Atrial Fibrillation: Comparing Uninterrupted Rivaroxaban and Warfarin (ASCERTAIN)**. *JACC Clinical electrophysiology* 2018, **4**(12):1598-1609.
42. Koretsune Y, Yamashita T, Kimura T, Fukuzawa M, Abe K, Yasaka M: **Short-Term Safety and Plasma Concentrations of Edoxaban in Japanese Patients With Non-Valvular Atrial Fibrillation and Severe Renal Impairment**. *Circulation journal : official journal of the Japanese Circulation Society* 2015, **79**(7):1486-1495.
43. Chung N, Jeon HK, Lien LM, Lai WT, Tse HF, Chung WS, Lee TH, Chen SA: **Safety of edoxaban, an oral factor Xa inhibitor, in Asian patients with non-valvular atrial fibrillation**. *Thrombosis and haemostasis* 2011, **105**(3):535-544.
44. Martischnig AM, Mehilli J, Pollak J, Petzold T, Fiedler AK, Mayer K, Schulz-Schüpke S,

Sibbing D, Massberg S, Kastrati A *et al*: **Impact of Dabigatran versus Phenprocoumon on ADP Induced Platelet Aggregation in Patients with Atrial Fibrillation with or without Concomitant Clopidogrel Therapy (the Dabi-ADP-1 and Dabi-ADP-2 Trials)**. *BioMed research international* 2015, **2015**:798486.

45. Kuwahara T, Abe M, Yamaki M, Fujieda H, Abe Y, Hashimoto K, Ishiba M, Sakai H, Hishikari K, Takigawa M *et al*: **Apixaban versus Warfarin for the Prevention of Periprocedural Cerebral Thromboembolism in Atrial Fibrillation Ablation: Multicenter Prospective Randomized Study**. *Journal of cardiovascular electrophysiology* 2016, **27**(5):549-554.
46. Wallentin L, Lopes RD, Hanna M, Thomas L, Hellkamp A, Nepal S, Hylek EM, Al-Khatib SM, Alexander JH, Alings M *et al*: **Efficacy and safety of apixaban compared with warfarin at different levels of predicted international normalized ratio control for stroke prevention in atrial fibrillation**. *Circulation* 2013, **127**(22):2166-2176.
47. Yamashita T, Koretsune Y, Yasaka M, Inoue H, Kawai Y, Yamaguchi T, Uchiyama S, Matsumoto M, Ogawa S: **Randomized, multicenter, warfarin-controlled phase II study of edoxaban in Japanese patients with non-valvular atrial fibrillation**. *Circulation journal : official journal of the Japanese Circulation Society* 2012, **76**(8):1840-1847.
48. Mao L, Li C, Li T, Yuan K: **Prevention of stroke and systemic embolism with rivaroxaban compared with warfarin in Chinese patients with atrial fibrillation**. *Vascular* 2014, **22**(4):252-258.
49. Sairaku A, Yoshida Y, Ando M, Hirayama H, Nakano Y, Kihara Y: **A head-to-head comparison of periprocedural coagulability under anticoagulation with rivaroxaban versus dabigatran in patients undergoing ablation of atrial fibrillation**. *Clinical drug investigation* 2013, **33**(11):847-853.
50. Ezekowitz MD, Reilly PA, Nehmiz G, Simmers TA, Nagarakanti R, Parcham-Azad K, Pedersen KE, Lionetti DA, Stangier J, Wallentin L: **Dabigatran with or without concomitant aspirin compared with warfarin alone in patients with nonvalvular atrial fibrillation (PETRO Study)**. *The American journal of cardiology* 2007, **100**(9):1419-1426.
51. Nin T, Sairaku A, Yoshida Y, Kamiya H, Tatematsu Y, Nanasato M, Inden Y, Hirayama H, Murohara T: **A randomized controlled trial of dabigatran versus warfarin for periablation anticoagulation in patients undergoing ablation of atrial fibrillation**. *Pacing and clinical electrophysiology : PACE* 2013, **36**(2):172-179.
52. Königsbrügge O, Quehenberger P, Belik S, Weigel G, Seger C, Griesmacher A, Pabinger I, Ay C: **Anti-coagulation assessment with prothrombin time and anti-Xa assays in real-world patients on treatment with rivaroxaban**. *Annals of hematology* 2015, **94**(9):1463-1471.
53. Watanabe T, Tachibana K, Shinoda Y, Minamisaka T, Fukuoka H, Inui H, Ueno K, Inoue S, Mine K, Hoshida S: **Difference in left atrial D-dimer level in patients with atrial fibrillation treated with direct oral anticoagulant**. *BMC cardiovascular disorders* 2021, **21**(1):487.
54. Sezai A, Taoka M, Osaka S, Kitazumi Y, Suzuki K, Kamata K, Tanaka M: **A Comparative Prospective Observational Study on the Use of Direct Oral Anticoagulants after**

Cardiac Surgery for the Management of Atrial Fibrillation. *Annals of thoracic and cardiovascular surgery : official journal of the Association of Thoracic and Cardiovascular Surgeons of Asia* 2021, **27**(3):191-199.

55. Pacholczak-Madej R, Bazan-Socha S, Zaręba L, Undas A, Dropi ń ski J: **Direct oral anticoagulants in the prevention of stroke in breast cancer patients with atrial fibrillation during adjuvant endocrine therapy: A cohort study**. *International journal of cardiology* 2021, **324**:78-83.
56. Patti G, Parato VM, Cavallari I, Calabrò P, Russo V, Renda G, Gragnano F, Pengo V, D'Onofrio A, Grimaldi M *et al*: **A Prospective Study to Evaluate the Effectiveness of Edoxaban for the Resolution of Left Atrial Thrombosis in Patients with Atrial Fibrillation**. *Journal of clinical medicine* 2022, **11**(7).
57. Li X, Zhang X, Jin Q, Xue Y, Lu W, Ge J, Zhou D, Lv Q: **Clinical Efficacy and Safety Comparison of Rivaroxaban and Dabigatran for Nonvalvular Atrial Fibrillation Patients Undergoing Percutaneous Left Atrial Appendage Closure Operation**. 2021, **12**.
58. Van Mieghem NM, Unverdorben M, Hengstenberg C, Möllmann H, Mehran R, López-Otero D, Nombela-Franco L, Moreno R, Nordbeck P, Thiele H *et al*: **Edoxaban versus Vitamin K Antagonist for Atrial Fibrillation after TAVR**. *The New England journal of medicine* 2021, **385**(23):2150-2160.
59. Yu Q, Chen C, Xu J, Xiao Y, Bao J, Yuan L: **Efficacy and safety evaluation of rivaroxaban vs. warfarin among non-valvular atrial fibrillation patients undergoing lower extremity revascularization**. *Frontiers in cardiovascular medicine* 2022, **9**:978639.
60. Frisullo G, Profice P, Brunetti V, Scala I, Bellavia S, Broccolini A, Caliandro P, Di Iorio R, Morosetti R, Pilato F *et al*: **Prospective Observational Study of Safety of Early Treatment with Edoxaban in Patients with Ischemic Stroke and Atrial Fibrillation (SATES Study)**. *Brain sciences* 2020, **11**(1).
61. Wang L, Yao W: **A Cohort Study on the Safety and Efficacy of Warfarin and Rivaroxaban in Anticoagulant Therapy in Patients with Atrial Fibrillation Study**. *BioMed research international* 2022, **2022**:4611383.
62. Okumura K, Akao M, Yoshida T, Kawata M, Okazaki O, Akashi S, Eshima K, Tanizawa K, Fukuzawa M, Hayashi T *et al*: **Low-Dose Edoxaban in Very Elderly Patients with Atrial Fibrillation**. *The New England journal of medicine* 2020, **383**(18):1735-1745.
